# Immunotoxin Therapies for the Treatment of Epidermal Growth Factor Receptor-Dependent Cancers

**DOI:** 10.3390/toxins8050137

**Published:** 2016-05-04

**Authors:** Nathan Simon, David FitzGerald

**Affiliations:** Biotherapy Section, Laboratory of Molecular Biology, Center for Cancer Research, National Cancer Institute, National Institutes of Health, 9000 Rockville Pike, 37/5124 Bethesda, MD 20892, USA; nathan.simon@nih.gov

**Keywords:** immunotoxin, EGFR, cancer therapeutic, clinical development

## Abstract

Many epithelial cancers rely on enhanced expression of the epidermal growth factor receptor (EGFR) to drive proliferation and survival pathways. Development of therapeutics to target EGFR signaling has been of high importance, and multiple examples have been approved for human use. However, many of the current small molecule or antibody-based therapeutics are of limited effectiveness due to the inevitable development of resistance and toxicity to normal tissues. Recombinant immunotoxins are therapeutic molecules consisting of an antibody or receptor ligand joined to a protein cytotoxin, combining the specific targeting of a cancer-expressed receptor with the potent cell killing of cytotoxic enzymes. Over the decades, many bacterial- or plant-based immunotoxins have been developed with the goal of targeting the broad range of cancers reliant upon EGFR overexpression. Many examples demonstrate excellent anti-cancer properties in preclinical development, and several EGFR-targeted immunotoxins have progressed to human trials. This review summarizes much of the past and current work in the development of immunotoxins for targeting EGFR-driven cancers.

## 1. Introduction

Cancers have become one of the primary causes of human morbidity and mortality, with worldwide estimates suggesting greater than 20 million new diagnoses annually within the next decade. Progression of many cancers is driven by the genetic alteration or overexpression of specific genes that upregulate signaling pathways involved in proliferation, metastasis, and survival. One group of genes prominently implicated in the development of multiple cancers is the epidermal growth factor receptor (EGFR/HER) genes, a family of four receptor tyrosine kinases (EGFR/HER2/HER3/HER4) involved in a variety of cell signaling pathways (reviewed in [1,2]). 

**Figure 1:**
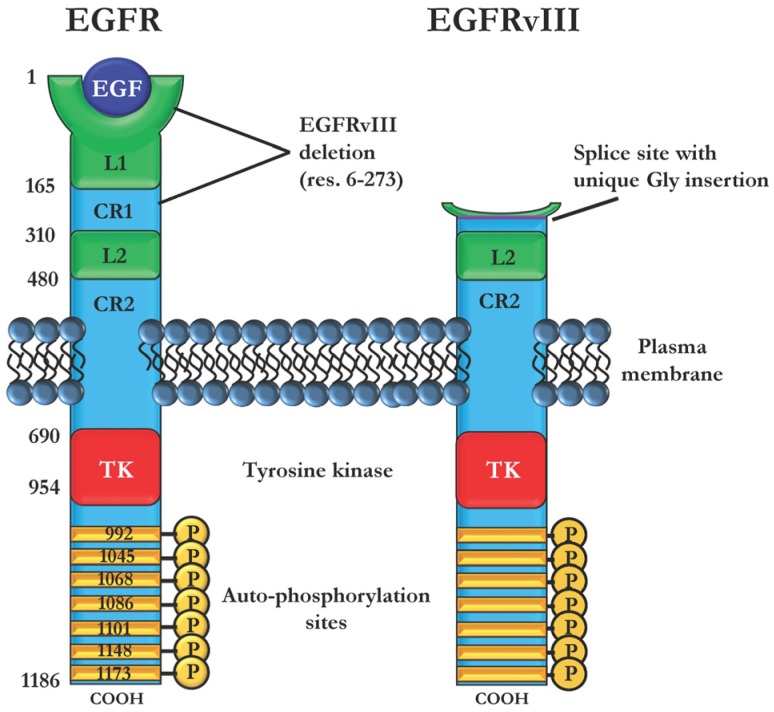
Structure–function organization of the epidermal growth factor receptor (EGFR) and EGFRvIII. EGFR consists of an extracellular ligand-binding region and an intracellular tyrosine kinase region. The extracellular component consists of two ligand binding domains (L1, L2) and two cysteine-rich regions (CR1, CR2) responsible for proper positioning of the ligand binding domains. Upon ligand binding, the receptor assumes an elongated “untethered” conformation and subsequently dimerizes with another EGFR. Upon dimerization, the tyrosine kinase (TK) domain becomes activated and autophosphorylates the receptor. The phosphorylated carboxy terminus becomes a docking site for downstream signaling proteins, which are themselves phosphorylated to promote signaling activation. Mutant EGFRvIII retains the intracellular architecture of EGFR; however, a deletion of residues 6–273 removes much of the ligand binding region. This mutation also results in a constituitively active kinase domain and hyperactive signaling.

The EGFR proteins consist of an extracellular ligand-binding domain, which binds various ligands including epidermal growth factor (EGF), transforming growth factor alpha (TGFα), and heparin binding-EGF (HB-EGF), and an intracellular ATP-binding kinase domain (Figure 1). Binding of ligand results in receptor homo- or heterodimerization among EGFR family proteins and activation of the tyrosine kinase domain. Upon activation and dimerization, the kinase domain autophosphorylates the carboxy-terminus of the receptor, allowing binding and activation of downstream signaling partners. These signaling proteins can activate cancer-promoting pathways like cell survival (JAK/STAT), proliferation (MAPK/ERK), angiogenesis (PI3K/AKT), and metastasis (PLC) [3]. EGFR mutations are commonly observed in cancers with both point mutations and large deletions observed in clinical cases. EGFR point mutations are associated with both sensitivity (L858R) and resistance (T790M) to tyrosine kinase inhibitor (TKI) therapies [4,5]. One of the most notable EGFR mutations is the variant III deletion (EGFRvIII) (reviewed in [6]). The deletion of EGFR exons 2–7 results in a protein with a truncated extracellular domain that eliminates ligand binding but gains constitutively active kinase activity (Figure 1) [7]. EGFRvIII expression is linked to glioblastoma insensitivity to chemotherapeutic agents through constitutive activation of survival pathways [8,9]. While EGFR activity is crucial to healthy cell functioning, dysregulation of receptor signaling events often leads to aberrant cell growth and development of malignancies. 

Mutation, amplification, or overexpression of the prototype member, EGFR (HER1/ErbB), occurs in breast, lung, bladder, head-and-neck, and pancreatic cancers. More than 60% of triple-negative breast cancers (TNBC) overexpress EGFR and increased expression strongly correlates with cancer progression and negative outcomes [10]. Ninety-percent of pancreatic cancers, which have a 5-year survival rate of less than 5%, display overexpression of EGFR or the EGFR ligands TGFα and EGF [11,12]. Non-small cell lung cancers (NSCLC) and head-and-neck cancers also show overexpression of EGFR and EGFR ligands in more than half of tumor samples [13,14]. Bladder cancers overexpress EGFR, while normal bladder epithelium expresses little or no EGFR [15,16] suggesting that these cancers may too be susceptible to an EGFR-directed therapy. Glioblastomas display EGFR overexpression in greater than 80% of samples and more than 50% display additional expression of EGFR deletion variant EGFRvIII [17,18]. Other EGFR family members, particularly HER2, have been implicated in colorectal, breast, ovarian, and gastric cancers, but will not be covered here (reviewed in [2,19]). Across a multitude of cancer types, enhanced activation and signaling from EGFR receptors correlate with increased cancer aggressiveness and poor patient outcomes (reviewed in [20,21]). 

With a wide spectrum of aggressive cancers and minimal therapeutic options, development of effective therapeutics against EGFR-expressing cancers has become a major field of study. A common approach for targeting the EGFR is through monoclonal antibodies targeting EGFR function. Several monoclonal antibodies that bind the EGFR extracellular domain and inhibit ligand binding or receptor dimerization have received full clinical approval for human use. Cetuximab (Erbitux®) is approved for treatment of colorectal and head-and-neck cancers, and panitumumab (Vectabix®) is approved for treatment of colorectal cancers [22]. However, newer therapeutics like matuzumab, nimotuzumab, or zalutumumab have shown less promise in clinical trials, and development of several next-generation examples has been discontinued due to little improvement in patient outcomes or adverse patient health effects [23,24,25,26]. Additional ligand-neutralizing antibodies targeting TGFα or HB-EGF have demonstrated anti-proliferative activity *in vitro* and inhibition of tumor growth and high dose-tolerability *in vivo* [27,28,29]. 

A second therapeutic mechanism for targeting EGFR activity is through the use of small molecule tyrosine kinase inhibitors that compete for the kinase active site to inhibit phosphorylation of downstream proteins. Multiple drugs have been clinically-approved, including gefitinib (Iressa®; NSCLC), lapatinib (Tykerb®; breast cancer), and erlotinib (Tarceva®; NSCLC and pancreatic cancers) [30,31,32,33]. However, patients treated with EGFR TKI invariably develop an EGFR kinase domain T790M “gatekeeper” mutation that blocks inhibitor access, rendering treatment effects only temporary [34]. Second and third generation TKI are being developed in an attempt to circumvent this mutation, and are undergoing human trials [35,36,37,38]. Additionally, while cells expressing EGFRvIII may be sensitive to EGFR TKIs, extended treatment results in downregulation of EGFR expression with no accompanying loss of oncogenic growth [39]. 

One major issue with using anti-EGFR therapeutics in a clinical setting is the potential for off-target effects of the therapeutic. Many healthy tissues have some level of EGFR expression, with the skin, liver, and gastrointestinal tracts expressing elevated levels of the protein. Inhibition of EGFR signaling in these healthy tissues by either anti-EGFR antibodies or TKI result in adverse effects, most commonly skin rash or gastrointestinal disorders. Between 50% and 100% of patients treated with anti-EGFR antibodies display various skin rashes, while diarrhea is the most common dose-limiting toxicity in patients treated with EGFR TKI [40]. Therefore, while targeting the activation or signaling of therapeutically-relevant proteins is often able to provide some anti-tumor activity, system-wide inhibition of important signaling pathways is undesirable. Additionally, treatment of the majority of cancers is hampered through therapy-driven genetic mutations or upregulation of alternative signaling pathways, suggesting that a mechanism that does not rely on direct inhibition of cellular signaling pathways would be of great use. 

**Figure 2:**
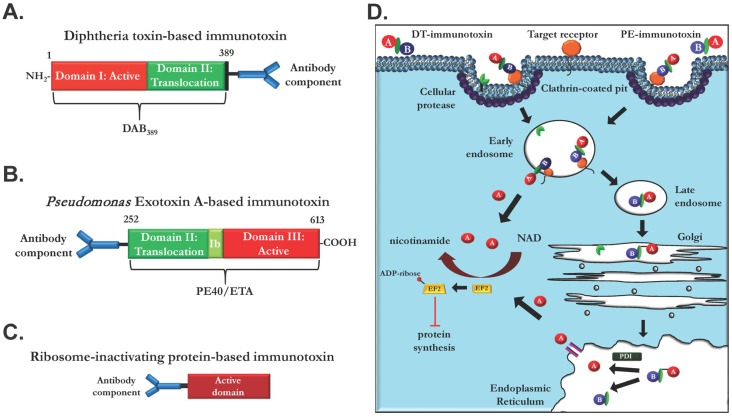
Immunotoxin domain organization and trafficking. (**A**) Immunotoxins derived from diphtheria toxin (DT) consist of cytotoxic ADP-ribosyltransferase domain I and translocation domain II, with receptor binding domain III replaced by the EGFR-targeting domain of interest. (**B**) Immunotoxins derived from *Pseudomonas* exotoxin (PE) are constructed in an inverse manner to DT. PE exotoxins consist of replacement of receptor binding domain I with a new targeting domain joined to the translocation (II) and ADP-ribosyltransferase (III) domains of PE. (**C**) Immunotoxins utilizing plant-derived ribosome-inactivating proteins (RIP; ricin, saporin, gelonin, dianthin) consist of the RNA glycosidase active domain chemically conjugated to the targeting moeity. (**D**) Upon endocytosis, immunotoxins enter varied trafficking pathways. Once internalized, cellular proteases cleave the peptide chain between active and translocation domains. DT-based immunotoxins are translocated to the cytosol upon endosomal acidification and disulfide reduction with no other trafficking required. Gelonin, saporin, and dianthin follow a similar pathway, although they do not require protease processing or possess translocation domains. PE-based immunotoxins enter a KDEL-mediated retrograde trafficking pathway, traveling through the Golgi apparatus to the endoplasmic reticulum. In the endoplasmic reticulum, disulfide reduction allows the cytotoxic domain to translocate to the cytosol with the help of ER-resident machinery. Ricin follows a similar pathway.

Antibody-cytotoxin fusions, or immunotoxins, have been under development for the treatment of cancers for several decades [41]. Historically, immunotoxins (IT) consist of an antibody or antibody fragment joined to a cytotoxin, typically a bacterial protein like diphtheria toxin (DT) (Figure 2A) or *Pseudomonas* exotoxin A (PE) (Figure 2B), or a plant-derived ribosomal inactivating protein (RIP) like ricin, gelonin, or saporin (Figure 2C) [42]. Immunotoxins can be engineered through either chemical conjugation of an antibody to the cytotoxin or through recombinant production of a fusion protein, joining an antibody, single chain Fv (scFv), or Fab to a protein toxin. Recombinant ITs most commonly consist of a gene fusion of the scFv of a relevant cell-targeting domain with the translocation and cell killing domains of DT or PE. As these therapeutics function by killing cells directly rather than through signaling inhibition, the possibility of escape mutation or upregulation of alternative signaling pathways is less of an issue. 

DT- and PE-based immunotoxins consist of the translocation and cytotoxic domains of the respective proteins with the toxin’s receptor binding domain replaced with the antibody, Fab, or scFv of interest (Figure 2A,B). The cytotoxic activity of DT and PE depends on the catalytic ADP-ribosylation of a unique diphthamide residue on elongation factor 2 (EF2) [43,44], resulting in inhibition of protein synthesis and induction of apoptosis in intoxicated cells [45]. Upon binding to the targeted cellular receptor, immunotoxins enter complex trafficking pathways involving multiple steps (Figure 2D). The targeted receptor is endocytosed and the cytotoxin domain is subsequently released from the targeting domain through furin-like protease activity and intra-chain disulfide reduction. Upon endosomal acidification, DT-based toxins are translocated into the cytosol where they find and ADP-ribosylate EF2 [46]. PE-based toxins possess a carboxy-terminal KDEL sequence and enter a retrograde trafficking pathway, passing through the Golgi to the endoplasmic reticulum. Once in the endoplasmic reticulum, the active toxin is translocated to the cytosol through an incompletely understood process and ADP-ribosylates EF2 [47]. EF2 ADP-ribosylation results in an inability to interact with the ribosome, resulting in termination of translation and eventual cell death. The enzymatic nature of these cytotoxins allows for high potency at low toxin concentrations, leading to estimates that delivery of a single molecule is sufficient to cause cell death [48]. Additionally, the limited size of recombinant immunotoxins compared to a full size antibody should allow greatly enhanced tumor penetration, and allow increased access to and killing of interior tumor cells [49].

Plant-derived cytotoxins like gelonin, saporin, and ricin function through a similar end reaction, inhibition of protein synthesis but use a unique catalytic mechanism. After binding to the target receptor and receptor internalization, saporin and gelonin require endosomal disruption to reach the cytosol [50]. Ricin is thought to follow a similar intoxication pathway to PE, trafficking in a retrograde fashion through the Golgi to the endoplasmic reticulum before translocation to the cytosol. Interestingly, the pathways may not be identical, as cells resistant to ricin-based immunotoxin are not necessarily resistant to an identical PE-based immunotoxin [51]. Once in the cytosol, RIPs remove a conserved adenosine from the 28S ribosomal RNA, blocking further translation and resulting in inhibition of protein synthesis in intoxicated cells. However, ricin has fallen out of favor as a cytotoxic moiety, as human trials with ricin-based immunotoxins show dose-limiting damage to the vasculature [52].

One potential issue with using plant- or bacterial-derived immunotoxins as human therapies is the generation of patient-derived neutralizing antibodies. Clinical trials have demonstrated that 88% of patients treated with the PE-based SS1P immunotoxin made anti-PE antibodies after one month of treatment [53]. However, additional work has shown that combination of immunotoxin treatment with immunosuppressant regimens reduces the formation of anti-toxin antibodies in mice [54] or humans [55]. Additionally, removal of toxin B-cell [56] and T-cell epitopes [57] has resulted in immunotoxins that do not stimulate development of anti-drug antibodies. These studies indicate that while immunogenicity may be an issue with immunotoxin therapy, it can be overcome with application of appropriate protein engineering and combination therapy strategies.

The ability to combine the specific cell targeting of therapeutic antibodies with the potent cell killing abilities of bacterial or plant proteins has necessarily led to the testing of immunotoxins in various clinical settings. DAB_389_IL2 (denileukin difitox), a fusion of the cytokine IL2 and DT, has been clinically approved for treating cutaneous T-cell lymphoma [58]. Phase I trials have demonstrated that the anti-mesothelin SS1P immunotoxin is well tolerated by human patients, and combination with current front-line chemotherapy has proven effective at treating advanced malignant mesothelioma with an overall response rate of 77% [53,59]. Additional combination with immunosuppressants further improves patient outcomes [55]. Treatment of patients with relapsed hairy cell leukemia with an anti-CD22 immunotoxin has resulted in durable long-term remissions in patients with the immunotoxin currently in Phase III testing [60]. The potency and adaptability of these therapeutics has led to much work designing an immunotoxin targeting the EGFR for use as a new front-line therapeutic. This review will examine past and recent work in this area, looking at the development of novel EGFR-targeted therapeutics from preclinical investigation (Table 1) to those entered in human trials (Table 2).

## 2. Literature

### 2.1. Ligand Immunotoxins

**Figure 3:**
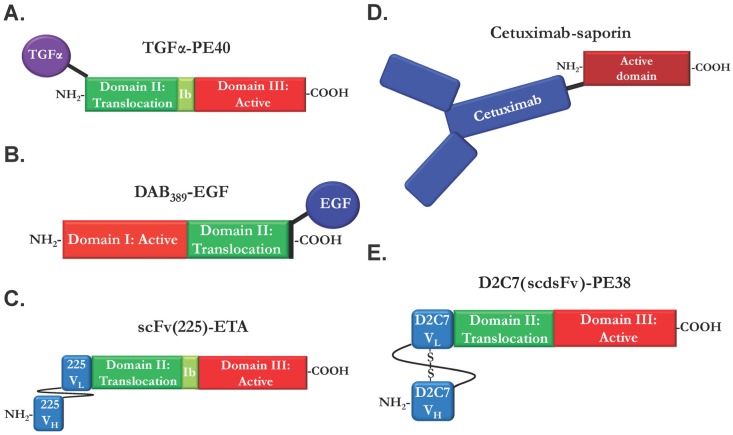
Anti-epidermal growth factor receptor immunotoxins possess considerable variability in domain organization and targeting moieties. (**A**) Transforming growth factor α-PE40 is the prototype anti-EGFR immunotoxin and an example of a ligand-immunotoxin [61]. TGFα-PE40 utilizes the EGFR ligand TGFα as a targeting component and domains II, Ib, and III of *Pseudomonas* Exotoxin A (PE) as the cytotoxin component. (**B**) DAB389-EGF functions similarly to TGFα-PE40 but uses epidermal growth factor (EGF) for the receptor ligand and swaps PE for a diphtheria toxin (DT)-based cytotoxin component [84]. (**C**) The majority of recent EGFR-targeted immunotoxins utilize a single chain Fv (scFv) from an anti-EGFR monoclonal antibody. A prototype molecule, scFv(225)-ETA, consists of the variable heavy (V_H_) and light (V_L_) chains of the monoclonal 225 antibody joined by a flexible peptide linker and fused to the PE40 cytotoxin derivative [63]. (**D**) Full-length IgG have been chemically conjugated to cytotoxins, as in the case of cetuximab-saporin [66]. These molecules retain bivalent binding, but their large size has led to questions about tumor penetration ability. (**E**) D2C7(scdsFv)-PE38 consists of the scFv of an antibody capable of binding both wild type EGFR and deletion mutant EGFRvIII [85]. Use of scFv allows for a smaller molecule, but the flexibility of the connection can result in loss of stability or binding. The introduction of an intra-chain disulfide linking V_H_ and V_L_ increases receptor affinity and immunotoxin stability.

EGFR, like other receptor tyrosine kinases, is activated through the binding of various extracellular ligands like TGFα and EGF. While antibodies targeting a cell surface receptor are the most common binding components of immunotoxins, the use of receptor ligands as the targeting moiety of the molecule has also been explored. Ligand binding to EGFR results in receptor dimerization and endocytic uptake, where the immunotoxin then follows defined trafficking pathways to the cytosol to exert toxic effects [78]. Initial ligand–immunotoxin work focused on targeting the EGFR with a fusion of TGFα with a 40 kDa fragment of PE consisting of the translocation and cytotoxic domains (TGFα-PE40/TP40) (Figure 3A) [61]. This molecule was tested on epidermoid and prostate cancer cells known to express amplification of EGFR, and proved to be highly active *in vitro* with IC_50_ (inhibitory concentration: 50% inhibition) lower than 1ng/ml and doubling the mouse survival time in murine xenograft models [79,80]. Later protein engineering led to TGFα immunotoxins with truncated PE domains of 38, 35 and 31 kDa, which were more potent than the parental TGFα-PE40 [81,82,83].

Bladder cancers rely upon TGFα as the primary EGFR ligand [86], suggesting that therapeutics utilizing TGFα as a targeting agent would be beneficial for treatment of these malignancies. Preclinical studies demonstrated that TGFα-immunotoxins are highly cytotoxic to bladder cancer cells and patient bladder cancer explants, despite the lack of EGFR amplification in these cells [87,88]. Transition of this molecule to Phase I clinical trials showed that TP40 is well tolerated in patients with superficial bladder cancer with no dose limiting toxicities noted [89]. Eight of nine patients with carcinoma *in situ* demonstrated partial or complete responses to treatment. Patients with invasive disease showed no response to treatment and no visible changes in tumors observed; however, this observation may be due to the therapeutic being delivered directly into the urinary bladder, while a systemic administration would potentially allow better treatment distribution. TP38, a similar molecule with TGFα linked to a 38 kDa fragment of PE, was tested in Phase I trials of malignant brain tumors with intracranial infusion techniques utilized for treatment delivery directly to tumor tissue [73,74]. Two of fifteen patients demonstrated radiographic responses to treatment with survivals of greater than 200 weeks post-treatment. However, two dose limiting neurologic toxicities were noted, including grade 3 hemiparesis and grade 4 fatigue. Additionally, the observation that >80% of infusions resulted in treatment leakage to other areas of the brain suggests that intracranial infusions may be less effective in practice. 

EGF-based immunotoxins have also received research attention. Initial development of a fusion of EGF to the catalytic domain of DT resulted in a protein that was non-toxic to EGFR-expressing cells *in vitro* [90]. Subsequent protein engineering confirmed that domain II, the “translocation domain”, of diphtheria toxin is required for endosome escape and cytotoxicity. A fusion of EGF and the catalytic and translocation domains of DT (DAB_389_EGF) (Figure 3B) killed multiple human cancers at pM concentrations [84]. Similar to TGFα-PE40, DAB_389_EGF has been investigated for the treatment of bladder cancers, demonstrating potent killing of bladder cancer cells *in vitro* and elimination of tumor burden in five of six mice *in vivo* [91]. This molecule has undergone additional preclinical investigation for treatment of brain cancers with amplification or overexpression of EGFR, showing cell killing abilities at pM concentrations against a panel of human glioblastoma cell lines [92]. In mice, DAB_389_EGF has a dose-limiting toxicity of renal failure at doses of 5 µg/mouse; however, 75% of mice implanted with glioblastoma and subsequently treated with 3 µg DAB_389_EGF /mouse showed tumor regression and were in remission at 60 days post-treatment [93]. Intracranial delivery of EGF-DT is also efficacious in a murine model of NSCLC brain metastasis, increasing survival time by 28% [94]. 

A Phase I/II clinical trial of DAB_389_EGF for treatment of solid tumors with EGFR expression enrolled fifty-two patients with metastatic disease who were treated with increasing doses of DAB_389_EGF [75]. One patient (NSCLC) displayed a partial response, and three others showed stable disease through the duration of the trial. However, all patients developed anti-DT or anti-EGF neutralizing antibodies, and dose-limiting toxicities due to liver or kidney damage and chest pain were observed. EGF or HB-EGF have also been conjugated to other toxin fragments, with EGF-PE [95], EGF-ricin [90], EGF-saporin [62], and HBEGF-saporin [96,97] also showing specific toxicity to EGFR-expressing cancers *in vitro* or *in vivo* while remaining mostly non-toxic to healthy cells. However, the lack of sustained clinical responses coupled with patient-generated neutralizing antibodies has hindered further clinical development of EGFR-ligand immunotoxins. 

### 2.2. Immunotoxins Based on Monoclonal EGFR Antibodies

EGFR-ligand immunotoxins are potent anti-EGFR therapeutics in a preclinical research setting, but have proven less successful in clinical trials due to dose-limiting toxicities and poor response rates. Many current anti-cancer therapeutics are based on monoclonal antibodies, which allow for greater targeting specificity and improved potency through protein engineering techniques [98]. Anti-EGFR antibodies have been under investigation for treatment of various EGFR-expressing cancers for several decades, with varying degrees of success [99,100,101,102]. Many researchers have taken advantage of the high affinity of these monoclonal antibodies for EGFR to build anti-EGFR immunotoxins, with the goal of delivering low levels of toxin to cells overexpressing the EGFR while sparing cells with low or normal levels of the protein. Most commonly, the scFv of the antibody is used as the binding component (Figure 3C), to retain much of the antibody’s affinity while rendering the therapeutic much smaller for better tumor penetration. However, antibody Fab or even entire antibody IgG have been used as EGFR targeting moieties (Figure 3D).

The mouse monoclonal antibody (MAb) 225 was identified in 1983 as a potent inhibitor of EGF binding to EGFR, resulting in a significant decrease in proliferation of A431 epidermoid carcinoma cells displaying EGFR amplification [103]. MAb225 not only inhibited ligand binding, but also promoted receptor internalization in the absence of receptor activation [104], suggesting it could be an ideal candidate for immunotoxin delivery. An immunotoxin constructed from the scFv of MAb225 fused to domains II and III of PE (scFv(225)-ETA) (Figure 3C) proved strongly cytotoxic to breast and epidermal cancer cells with amplified EGFR [63]. scFv(225)-ETA inhibited EGFR activation in the presence of EGF, suggesting that this immunotoxin can inhibit EGFR activity either by interrupting signaling or through cytotoxic activity. scFv(225)-ETA exhibited cytotoxicity towards cells with even moderate EGFR expression, but showed no toxicity towards cells with no EGFR expression [105]. Treatment of CAL27 squamous cell xenografts with scFv(225)-ETA resulted in significant tumor growth suppression, although no complete regressions were noted for this treatment.

Another early antibody was identified to strongly bind the EGFR was the monoclonal anti-EGFR 425 antibody (MAb425) [106]. MAb425 binds an epitope distinct from that bound by MAb225/cetuximab [107], but still possesses anti-tumor activity *in vivo* [108]. The MAb425 scFv has been engineered into an immunotoxin, 425(scFv)-ETA, comprised of the MAb425 scFv fused to the 40 kDa fragment of PE consisting of the translocation and cytotoxic domains (PE40/ETA). 425(scFv)-ETA was strongly cytotoxic towards metastatic pancreatic cancer cells with an IC_50_ of less than 10 ng/mL [64], while EGFR-negative cells were unaffected at concentrations of immunotoxin lower than 10 µg/mL. 425(scFv)-ETA was also highly effective against pancreatic tumors metastases *in vivo.* In mice injected with metastatic pancreatic cells, multiple injections of 425(scFv)-ETA reduced the number of lung metastases from 56 per mouse to 0.28 per mouse, suggesting that immunotoxins may be effective at targeting not only primary tumors but also secondary tumor spread [109]. Furthermore, 425(scFv)-ETA bound *ex vivo* tissue from patients with rhabdomyosarcoma, an aggressive cancer most common in children, and killed rhabdomyosarcoma cells with pM potency *in vitro* [110]. MAb425 has been humanized (matuzumab), but Phase II human trials did not result in significantly improved outcomes [25] and development has been halted, suggesting that 425(scFv)-ETA may not be an ideal candidate for further development.

While immunotoxins derived from mouse antibodies possess considerable preclinical activity, further development would require antibody humanization and human safety profiling. Taking advantage of molecules with established safety profiles, several groups have investigated the use of antibodies already clinically approved as targeting moieties. Anti-EGFR antibodies approved for use in treating human cancers include cetuximab, approved for colorectal and head-and-neck cancers, and panitumumab, approved for colorectal cancers. Cetuximab is a human-mouse chimeric protein consisting of the mouse MAb225 variable region and a humanized constant region and has 10-fold higher affinity for EGFR than the parental MAb225 [111]. While panitumumab is a fully humanized antibody [112], both antibodies bind a similar epitope on the EGFR extracellular domain and function through inhibition of ligand binding [113]. With knowledge that the targeting domains are safe for human use, ITs utilizing the full-length antibody or antibody fragments of cetuximab or panitumumab have received increasing attention in preclinical studies.

The immunotoxins scFv2112-ETA and scFv1711-ETA are composed of the scFv of cetuximab and panitumumab, respectively, joined to domains II and III of PE (PE40/ETA) [65]. These immunotoxins were tested *in vitro* and *ex vivo* against a panel of cancer types known to rely on EGFR signaling, including breast, epidermoid, pancreatic, and prostate cancers. Both immunotoxins demonstrated strong induction of apoptosis and potent cell killing toward representative cell lines with a range of EGFR expression from 149,000 receptors/cell to 12,500 receptors/cell. Interestingly, scFv2112-ETA was more toxic to epidermoid, breast, and prostate cancers, while scFv1711-ETA was more toxic to rhabdomyosarcoma and pancreatic cancers. Cytotoxic potency ranged from 4–460 pM and correlated with EGFR expression. EGFR-null cells were unaffected, confirming the specificity of these immunotoxins toward EGFR-expressing cancers. Notably, both immunotoxins stimulated apoptosis in a greater percentage of cells than cetuximab or panitumumab alone. *Ex vivo* studies demonstrated strong binding of both constructs to tissue samples obtained from patients with breast, prostate, and rhabdomyosarcoma cancers. With their safety in humans already established, the use of previously approved antibodies for IT construction could be more effective and less immunogenic in clinical cancer treatment.

One property of monoclonal antibody therapeutics that most immunotoxins do not possess is the induction of antibody-dependent cell-mediated cytotoxicity (ADCC), which relies on the Fc component of the antibody being recognized by natural killer (NK) cells. Upon Fc binding, the NK cells become activated and stimulate killing of antibody-bound tumor cells [114,115]. Preservation of this additional cell killing modality in immunotoxin treatment has been attempted through the use of the full length cetuximab IgG as an immunotoxin targeting component [66]. The full-length cetuximab IgG was covalently conjugated to the plant ribosome-inactivating protein saporin, creating the cetuximab-saporin immunotoxin (Figure 3D). On its own, cetuximab-saporin possesses low cytotoxicity, with 10 nM concentrations failing to kill cells. However, the addition of saponins, plant-derived chemicals which specifically create pores in endosomes/lysosomes [116], increases immunotoxin potency by up to 1000-fold with no effects on non-target cells [117]. Upon addition of the saponin SO1861, cetuximab-saporin demonstrated complete cell killing at a 10 nM concentration, suggesting that an inability of the saporin to access the cytosol was to blame for the poor cytotoxicity [66]. In addition to the saporin cytotoxic activity, retention of the Fc of the full cetuximab antibody stimulated NK-cell dependent ADCC, enhancing the cell killing potential of cetuximab-saporin. 

Similar results have been reported for panitumumab-dianthin and cetuximab-dianthin immunotoxins. Dianthin is another plant-derived ribosome-inactivating protein, cleaving an adenosine from ribosomal RNA to prevent protein synthesis [118]. Dianthin-panitumumab and dianthin-cetuximab immunotoxins engineered from the full length therapeutic IgG conjugated to recombinant dianthin similarly showed no killing of EGFR-overexpressing colorectal cancer cells when administered at concentrations below 10 nM [67]. Addition of the SO1861 saponin significantly increased the cytotoxicity of these immunotoxins with IC_50_ values of 1.5 pM (dianthin-panitumumab) and 5.3 pM (dianthin-cetuximab). 

The potency and non-bacterial origin of IgG-RIP immunotoxins suggests that they may be suitable for further clinical development. To date, no *in vivo* results have been reported for cetuximab or panitumumab-based immunotoxins; however, two potential issues may limit the potency of these constructs *in vivo*. The lack of an efficient cellular trafficking pathway for these toxins, with requirement for endosome/lysosome disruption by saponins, may not allow the therapeutics to reach peak toxicity. The size of full IgG immunotoxins, more than 200 kDa, may hamper the ability to sufficiently penetrate into solid tumors resulting in poorer treatment [49]. Further experiments *in vivo* should shed more light on the therapeutic potential of these RIP-based immunotoxins.

### 2.3. Immunotoxins Targeting Cancers Expressing Mutant EGFRvIII

Immunotoxins targeting wild type EGFR may be ideal for treating cancers with EGFR amplification or overexpression, but some EGFR-driven cancers rely upon expression of EGFRvIII, a truncated form of EGFR. EGFRvIII is a splice variant in which a mutation causing exons 2–7 to be removed results in a deletion of 801 DNA base pairs corresponding to residues 6–273 of the extracellular domain of the protein (Figure 1). This in-frame deletion also results in the creation of a unique glycine residue and unique peptide sequence at the splice site [119]. The loss of a portion of the extracellular protein renders EGFRvIII incapable of binding ligands. Instead, EGFRvIII gains constitutive kinase activity, resulting in hyperactivation of EGFR signaling and enhanced proliferation and survival over cancers with EGFR alone [120,121]. EGFRvIII is not expressed in healthy tissues, indicating that it is a cancer-specific marker and making it an ideal therapeutic target. EGFRvIII is most commonly noted in glioblastoma (40%–60%) [17,122] and is detected rarely in lung cancers [123], head-and-neck cancers [124] or breast cancers [125]. In glioblastoma, which has five-year survival rates of less than 5% [126], EGFRvIII may be used as a prognostic factor as expression is significantly correlated with poor survival rates [127]. As treatment modalities for glioblastoma are currently limited to chemotherapy and radiation, the targeting of EGFRvIII with immunotoxins has received much attention. 

One challenge to creating an EGFRvIII-targeted immunotoxin is creating a binding fragment that will not have off-target reactivity with wild type EGFR on healthy cells. One approach that has proven successful in generating anti-EGFRvIII antibodies is the use of phage-display libraries. The L8A4 antibody, which targets the unique peptide formed by insertion of the novel glycine residue at the mutation splice site, was isolated from one such library [17]. A PE-based immunotoxin attached to the full-length L8A4 IgG was tested on EGFR-null N6M cells expressing EGFRvIII cDNA. As EGFRvIII is not maintained by cells in culture, much of the pre-clinical work on this protein required transient or stable expression of the protein from a plasmid [128]. L8A4-PE35 is cytotoxic to cells expressing EGFRvIII at pM concentrations, but only weakly toxic to cells overexpressing wild type EGFR [68]. However, therapeutics with large molecular weights have significantly worse tumor penetration than smaller molecules, so development of a similar immunotoxin with a smaller binding domain was desired for further work [49].

To identify an antibody fragment with similar binding specificity to L8A4, the unique peptide formed by the EGFRvIII splice variant was used as an antigen to generate a library of phage displaying murine scFv [69]. The scFv MR1 was isolated from this screen and was fused to the PE38 exotoxin to create MR1(Fv)-PE38 for treatment of glioblastoma expressing EGFRvIII. MR1(Fv)-PE38 was effective at killing glioblastoma cells both *in vitro* and *in vivo*. In a rat model of glioblastoma, MR1(Fv)-PE38 immunotoxin treatment extended animal median survival to >53 days, compared to control animal survival of merely seven days [129]. Improvements to the binding affinity of MR1 were made through targeted mutagenesis of the complementary determining region-3 of the heavy and light chains of the scFv [130]. The resulting MR1-1 possesses 15-fold higher affinity for EGFRvIII, demonstrating the strength of antibody affinity maturation [131]. MR1-1(Fv)-PE38 displayed 3.5-fold increased potency toward cells expressing EGFRvIII compared to the parental MR1(Fv)-PE38 [130]. 

An issue with treating diseases of the brain is delivering large molecular weight therapeutics across the blood-brain barrier. One technique developed to circumvent this issue is convection enhanced delivery (CED), in which a drug is delivered directly to the brain tissue through a catheter and circulated throughout with the use of pressure gradients [132]. As immunotoxins are large therapeutics, a pilot safety study utilizing CED to deliver MR1-1(Fv)-PE38 directly to tumor tissue was performed in rats [133]. All animals survived the treatment with no signs of neurotoxicity or other adverse effects noticed. MR1-1(Fv)-PE38 entered Phase I trials for safety profiling in treatment of malignant brain tumors; however, this trial has since been terminated due to low accrual [76]. Given the poor prognosis for current glioblastoma treatments, targeting EGFRvIII with immunotoxin therapy may be a viable alternative, although additional clinical work to determine ideal combination therapies and treatment approaches is necessary.

### 2.4. Bispecific and Conformation-Dependent Immunotoxins

Protein engineering techniques allowing for engineering of antibodies with specific properties have led to the “designing” of antibodies with the ability to target specific epitopes of a target protein. Utilizing the extracellular region common to both truncated EGFRvIII and wild type EGFR as an antigen, antibodies that can bind both EGFRvIII and wild-type EGFR are being used as the basis of bi-specific immunotoxins to target diverse cancers with either EGFR amplification or EGFRvIII expression. One example, the D2C7 antibody, was generated by immunizing mice with a peptide corresponding to the junction created by the EGFRvIII mutation [85]. D2C7 binds both EGFRvIII and EGFR and possesses the ability to bind malignant tissue that either overexpresses EGFR or expresses EGFRvIII. As glioblastomas often both overexpress EGFR and express EGFRvIII, this binding specificity has led to D2C7 being utilized as the basis of an immunotoxin currently being investigated for the treatment of these cancers. D2C7(scdsFv)-PE38KDEL is constructed from a disulfide-stabilized scFv of the D2C7 antibody fused to the 38 kDa fragment of PE with a carboxy-terminal KDEL sequence to enhance intracellular retrograde trafficking (Figure 3E). Stabilization of the scFv through engineering of a disulfide bridge is thought to improve both scFv binding affinity and stability. D2C7(scdsFv)-PE38KDEL was highly cytotoxic to glioblastoma cell lines expressing EGFRvIII, as well as non-glioblastoma cell lines with EGFR amplification. Treatment of mice implanted with intracranial EGFRvIII-expressing tumors with D2C7(scdsFv)-PE38KDEL enhanced survival by more than 150% [134]. Additional studies demonstrated enhanced binding to cancers with EGFR amplification compared to commercial anti-EGFR antibodies and minimal binding to non-tumor tissues in a murine distribution bioassay [85]. These studies have resulted in D2C7(scdsFv)-PE38KDEL receiving Investigational New Drug status and moving to clinical testing [135] in a Phase I/II study to determine maximum-tolerated dose and initial effectiveness in patients with malignant glioma [77]. 

The 806 monoclonal antibody (m806) has similar properties to D2C7, originally raised in mice against an antigen composed of the unique peptide generated by the EGFRvIII deletion [136]. The m806 antibody binds strongly to cells expressing EGFRvIII, but it also has a novel tumor-specific binding tropism. The 806 antibody only binds cells with overexpression or amplification of EGFR, without binding to cells expressing wild-type EGFR [137]. This unique property is the result of m806 binding a conformational epitope that is masked in inactive or dimerized EGFR and only becomes exposed in the “untethered” form of the receptor [138,139]. This transient activated form preferentially occurs in oncogenic cells with enhanced EGFR activation, so m806 binds cells with normal EGFR signaling at very low levels [140]. This unique property suggests it is an ideal candidate for targeting oncogenic cells without off-target effects on healthy tissues. With that in mind, several 806-based immunotoxins have been examined in preclinical studies. 

**Figure 4:**
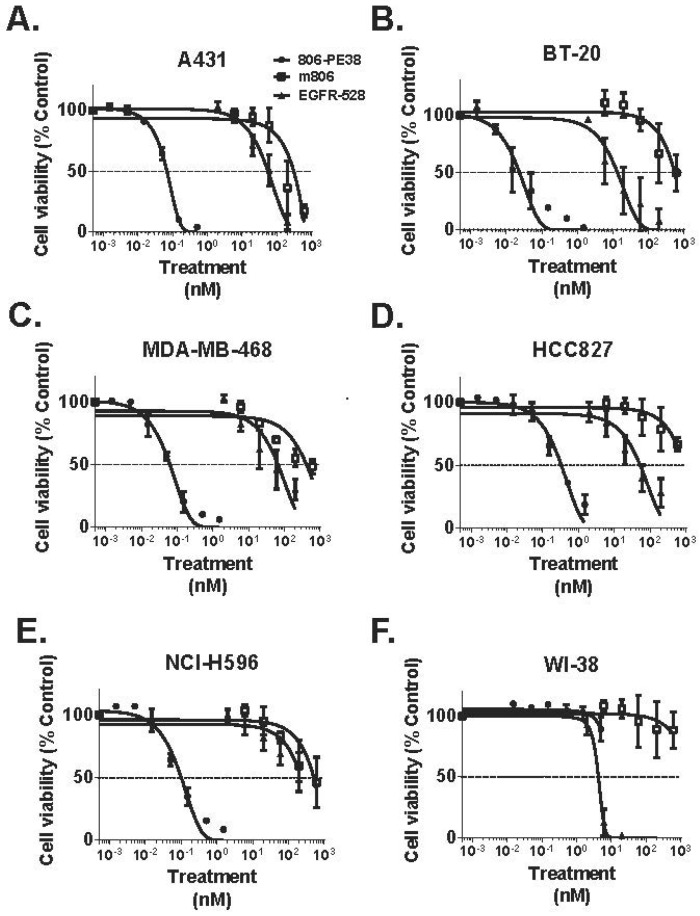
The 806-PE38 immunotoxin is 1000-fold more potent to malignant cells with EGFR overexpression than anti-EGFR antibodies. (**A**) A431 epidermoid carcinoma (**B**) BT-20 invasive ductal breast carcinoma (**C**) MDA-MB-468 triple-negative breast adenocarcinoma (**D**) HCC827 lung adenocarcinoma (**E**) NCI-H596 lung adenosquamous carcinoma or (**F**) WI-38 non-malignant epithelial cells were incubated with the indicated concentrations of 806-PE38 (●), the parental m806 antibody (□), or the anti-EGFR(528) antibody (▲) for 72 h. Cell viability was then measured with the Cell-Titer Glo assay. Data was normalized to a no-toxin control. Each assay was performed in triplicate at least twice.

DT390-BiscFv806 contains a bivalent single chain 806 FV fused to the ADP-ribosyltransferase and translocation domains of DT. This immunotoxin inhibited the viability of U87MG glioblastoma cells ectopically overexpressing EGFRvIII, as well as a panel of head and neck cancers with EGFR overexpression [71]. 806-PE38 was engineered in a similar fashion, but only contains a single 806 Fv valency and utilizes the PE38 derivative as the cytotoxin component. Preclinical studies demonstrated 806-PE38 inhibition of the viability of triple-negative breast cancers, which commonly show amplification or overexpression of EGFR, at concentrations below 150 pM [70]. 806-PE38 was similarly potent *in vivo*, enhancing the survival of mice implanted with TNBC xenografts by two-fold. 806-PE38 also was strongly cytotoxic toward epidermoid, breast, and lung cancers with EGFR amplification. 806-PE38 displayed 1000-fold higher inhibition of cell viability over the parental m806 antibody or an anti-EGFR antibody, demonstrating the increase in anti-cancer potency offered by immunotoxin cytotoxic activity over signaling inhibition alone (Figure 4). 806-PE38 was non-toxic to the non-malignant WI-38 epithelial cell line, confirming that the 806 antibody does not target non-malignant cells with normal EGFR expression.

A final example of anti-EGFR immunotoxin with the ability to bind both wild type EGFR and EGFRvIII is scFv(14E1)-ETA, consisting of the scFv of the 14E1 monoclonal antibody. 14E1 was isolated from mice immunized with A431 epidermoid carcinoma cells as an EGFR-binding antibody with nine-fold higher affinity for the extracellular domain of EGFR than cetuximab [72]. scFv(14E1)-ETA displayed similar potency as TGFα-PE38 against multiple cancer cell lines with EGFR overexpression. Unlike TGFα-PE38, however, scFv(14E1)-ETA was not toxic to cells without EGFR overexpression, suggesting it may not target healthy cells with normal EGFR levels. scFv(14E1)-ETA also binds EGFRvIII, displaying 100-fold more potency towards cells with EGFRvIII expression than cells with wild type EGFR [141]. In confirmation of this dual-targeting, scFv(14E1)-ETA proved to be effective at killing glioblastoma cells displaying resistance to both cetuximab and EGFR TKI [142]. Intriguingly, the commonly used chemotherapeutic cisplatin appears to upregulate EGFR in cisplatin-resistant glioblastoma [142]. Co-administration of cisplatin and scFv(14E1)-ETA was significantly more effective at killing the chemotherapy-resistant cells than either treatment alone. In preclinical studies of breast cancer patients, scFv(14E1)-ETA eliminated breast cancer cells from hematopoietic progenitor cell grafts *ex vivo* and was cytotoxic to 5/5 patient breast cancer tissue explants [143]. scFv(14E1)-ETA also demonstrated anti-metastatic capabilities, eliminating pulmonary metastases in an immunocompetent mouse model of metastatic renal cancer; however, all mice developed anti-PE antibodies resulting in neutralization of further toxin activity [144]. The ability to use a single therapeutic for cancers expressing either wild type EGFR or EGFRvIII broadly expands the range of cancers for which these molecules could become therapeutic options someday.

### 2.5. Photochemical Delivery of Anti-EGFR Immunotoxins

One potential issue with the use of EGFR-targeted immunotoxins is their necessity for delivery to the cytosol to exert their cytotoxic functions. During normal EGFR trafficking, once activated, the receptor is internalized and either recycled to the cell surface or sent to the lysosome for degradation. However, if the therapeutic does not efficiently escape the endosome and is recycled out to the cell surface or degraded in the lysosome, treatment efficacy would be severely compromised. To circumvent errant immunotoxin delivery, the development of a photochemical internalization (PCI) system to prematurely lyse IT-containing vesicles to deliver the therapeutic directly to the cytosol has been explored for several EGFR-targeted immunotoxins. In PCI, accumulation of a photosensitizer molecule in endosomes and lysosomes was followed by excitation of these molecules through targeted application of light to the tumor results in vesicle rupture, delivering the contents directly to the cytosol [145]. 

Proof-of-concept experiments demonstrated that toxicity of an EGF-saporin conjugate is enhanced more than 1000-fold when combined with PCI in various malignant cell lines with EGFR expression, while non-toxic to cells without EGFR [146]. Cetuximab possesses a two-log higher affinity for EGFR than EGF, so a similar construct made through the chemical conjugation of the full-length cetuximab antibody to saporin was also examined. Cetuximab-saporin was toxic to colon, prostate, and epithelial cancer cells, and combination with PCI resulted in significantly increased toxicity in all EGFR-expressing cell lines tested [147]. While the use of PCI with EGFR-targeted immunotoxins is relatively early in development, PCI has been used *in vivo* to enhance other immunotoxin activity with complete remissions observed in a mouse model of melanoma [148]. Similar in application to saponins, it is expected that the addition of PCI to immunotoxin therapy would allow for more effective treatment with fewer off-target effects. The increased bioavailability of active molecules escaping degradation may allow for decreased concentrations of potent therapeutics. When coupled with increased treatment specificity through light application to the tumor site alone, a decrease in off-target effects on non-cancerous tissues can be expected [149]. Future development of PCI in combination with EGFR-targeted immunotoxins may provide significant clinical benefits for patients with light-accessible tumors.

## 3. Conclusions 

Overexpression or amplification of the EGFR generally results in cancers with increased aggressiveness, higher rates of metastases, and worse patient prognosis. With the wide variety of cancers displaying overexpression of wild type EGFR or its mutants, it is of little surprise that so much work has gone into development of a specific and potent anti-EGFR therapeutic. While some success has been achieved in developing potent and lasting inhibition of these cancers, many current treatments prove too limited in duration. The combination of a potent enzymatic cytotoxin with an interchangable cellular targeting domain, unique properties of immunotoxins, explains why these molecules are perfectly suited for future anti-cancer development. Targeting of EGFR or EGFRvIII with various antibodies as a delivery vector for apoptosis-inducing cytotoxins has proven extremely successful in preclinical studies; however, clinical work to date has proven mostly unsuccessful. Increases in antibody and protein engineering techniques allowing for higher affinity binding domains and the removal of immune-stimulating epitopes may yet lead to powerful and safe therapeutics for the treatment of these cancers.

## Figures and Tables

**Table 1:** Pre-clinical development of anti-epidermal growth factor receptor.

Immunotoxin	Targeting Moiety	Toxin Moiety	Susceptible Cancer cell Types	Ref.
TGFα-PE40	TGFα; chemical conjugation	PE40	Epidermoid; prostate	[61]
EGF-saporin	EGF; chemical	saporin	Colon, prostate,epithelial	[62]
scFv(225)-ETA	Anti-EGFR MAb225 scFv; fusion protein	PE40	Breast; epidermoid; oral	[63]
425(scFv)-ETA	Anti-EGFR MAb425 scFv; fusion	PE40	Pancreatic; RMS	[64]
scFv2112-ETA	Cetuximab scFv; fusion	PE40	Epidermoid; breast; prostate	[65]
scFv1711-ETA	Panitumumab scFv; fusion	PE40	RMS; pancreatic	[65]
Cetuximab-saporin	Cetuximab IgG; chemical	saporin	-	[66]
Cetuximab-dianthin	Cetuximab IgG; chemical	dianthin	-	[67]
Panitumumab-dianthin	Panitumumab IgG; chemical	dianthin	-	[67]
L8A4-PE35	Anti-EGFRvIII L8A4 IgG; chemical	PE35	EGFRvIII-transfected N6M	[68]
MR1(Fv)-PE38	Anti-EGFRvIII MR1 scFv; fusion	PE38	Glioblastoma	[69]
806-PE38	Anti-EGFR/EGFRvIII MAb806 scFv; fusion	PE38	TNBC; NSCLC; Epidermoid	[70]
DT390-BiscFv806	Anti-EGFR/EGFRvIII MAb806 scFv; fusion	DT_390_	Head-and-neck; EGFRvIII-transfected U87MG	[71]
scFv(14E1)-ETA	Anti-EGFR/EGFRvIII MAb14E1 scFv; fusion	PE40	Glioblastoma; breast; renal	[72]

Abbreviations: TGFα: Transforming growth factor alpha; EGF: Epidermal growth factor; EGFR: Epidermal growth factor receptor; RMS: rhabdomyosarcoma; TNBC: Triple-negative breast cancer; NSCLC: Non-small cell lung cancer; PE40: Domains II (translocation), Ib, and III (cytotoxic) of *Pseudomonas* exotoxin; DT_390_: Domains I (cytotoxic) and II (translocation) of diphtheria toxin; PE38: Domains II (translocation) and III (cytotoxic) of *Pseudomonas* exotoxin.

**Table 2:** Clinical development of anti-epidermal growth factor receptor immunotoxins.

Immunotoxin	Targeting Moiety	Toxin Moiety	Clinical Trials: Indicated Cancer(s)	Clinical Trial Status	Ref.
TP38	TGFα; chemical conjugation	PE38	Malignant brain tumors	Phase I; discontinued	[73,74]
DAB_389_-EGF	EGF; chemical	DT_389_	Solid tumors with EGFR overexpression	Phase I/II; discontinued	[75]
MR1-1(Fv)-PE38	Anti-EGFRvIII MR1 scFv; fusion protein	PE38	Malignant brain tumors	Phase I; discontinued	[76]
D2C7(scdsFv)-PE38	Anti-EGFR/EGFRvIII MAbD2C7 scFv; fusion	PE38	Malignant glioma	Phase I/II	[77]

Abbreviations: Transforming growth factor alpha: TGFα; Epidermal growth factor: EGF; Epidermal growth factor receptor: EGFR; DT_389_: Domains I (cytotoxic) and II (translocation) of diphtheria toxin; PE38: Domains II (translocation) and III (cytotoxic) of *Pseudomonas* exotoxin.

## Abbreviations

The following abbreviations are used in this manuscript:

EGFR: epidermal growth factor receptor
IT: immunotoxin
scFv: single-chain variable fragment
PE: Pseudomonas exotoxin
DT: diphtheria toxin
EGF: epidermal growth factor
TGFα: transforming growth factor alpha
HB-EGF: heparin binding-EGF
TKI: tyrosine kinase inhibitor
TNBC: triple-negative breast cancer
NSCLC: non-small cell lung cancer
EF2: elongation factor 2
RIP: ribosome-inactivating proteins
MAb: monoclonal antibody
NK cell: natural killer cell
CED: convection enhanced delivery
PCI: photochemical internalization
